# Association Between Cortical Cerebral Microinfarct and Motor Performance at One-Year Follow-Up in Patients with Cerebral Small Vessel Disease: An Exploratory Study

**DOI:** 10.3390/brainsci16080892

**Published:** 2026-08-20

**Authors:** Dongyang Zhou, Hongyi Yan, Lei Guo, Shuo Yang, Tingting Wang, Ling Guan, Yilong Wang

**Affiliations:** 1 Department of Neurology, Beijing Tiantan Hospital, Capital Medical University, Beijing 100070, China; dyzhou15@163.com (D.Z.); yanhongyi1221@163.com (H.Y.); guolei53@aliyun.com (L.G.); capchisy@163.com (S.Y.); wangtingtingdr@sina.com (T.W.); 2 Department of Neurology, The First Affiliated Hospital of Zhengzhou University, Zhengzhou 450052, China; 3 Chinese Institute for Brain Research, Beijing 100070, China; 4 National Center for Neurological Disorders, Beijing 100070, China; 5 Advanced Innovation Center for Human Brain Protection, Capital Medical University, Beijing 100070, China; 6 China National Clinical Research Center for Neurological Diseases, Beijing 100070, China; 7 Beijing Laboratory of Oral Health, Capital Medical University, Beijing 100070, China; 8 Laboratory for Clinical Medicine, Capital Medical University, Beijing 100070, China

**Keywords:** cortical cerebral microinfarct, motor function, gait and balance disorders, cerebral small vessel disease

## Abstract

**Highlights:**

**What are the main findings?**

- Patients with CCMI showed poorer gait and balance performance at baseline.
- CCMI was associated with slower gait speed at one-year follow-up in patients with CSVD.

**What are the implications of the main findings?**

- CCMI may be related to slower gait in patients with CSVD.
- Larger longitudinal cohorts are needed to confirm the relationship between CCMI and gait function.

**Abstract:**

**Background/Objectives**: Cerebral small vessel disease (CSVD) is an important contributor to motor impairment. Cortical cerebral microinfarct (CCMI) is an emerging imaging marker of CSVD. Although its association with cognitive impairment has been well established, its relationship with motor function remains unclear. We explored associations of CCMI with motor performance at baseline and 1-year follow-up in this secondary analysis of data from the China Imaging-based Biobank of Cerebral Small Vessel Diseases. **Methods**: CCMI was assessed on baseline brain magnetic resonance imaging. Motor function was assessed at baseline and at 1-year follow-up. Outcomes were gait speed, poor balance performance (Short Physical Performance Battery balance score ≤ 2), abnormal gait (Scale for the Assessment and Rating of Ataxia gait score ≥ 2), and repeated chair-stand time. Gait speed was the primary outcome and all other motor outcomes were secondary. Multivariable linear and logistic regression models were used, with Benjamini–Hochberg correction applied to secondary outcomes. **Results**: The analysis included 192 patients, of whom 74 had 1-year follow-up motor data. At baseline, 55 patients (28.65%) had CCMI. Compared with patients without CCMI, those with CCMI had lower gait speed [0.92 (0.75–1.12) vs. 1.05 (0.87–1.16) m/s]. They also had higher prevalences of poor balance performance (29.09% vs. 14.60%) and abnormal gait (63.64% vs. 36.50%). However, fully adjusted analyses provided no conclusive evidence of baseline associations. At 1-year follow-up, CCMI was associated with slower gait speed after adjustment for demographic factors, baseline gait speed, and total CSVD burden (adjusted β −0.16, 95% CI −0.24 to −0.08; *p* < 0.001). CCMI was also nominally associated with longer repeated chair-stand time (adjusted β 2.07, 95% CI 0.39–3.75; nominal *p* = 0.02; adjusted *p* = 0.06), but this association did not survive correction for multiple comparisons. **Conclusions**: CCMI was associated with slower gait speed at 1-year follow-up in patients with CSVD. This finding is hypothesis-generating and requires confirmation.

## 1. Introduction

Cortical cerebral microinfarct (CCMI) is a small ischemic lesion confined to the cerebral cortex and has increasingly been recognized as a clinically relevant marker of cerebral small vessel disease (CSVD). CCMI has been associated with cognitive impairment, dementia, and other adverse neurological outcomes [1,2,3]. Neuropathological studies have reported cerebral microinfarcts in approximately 24% of older adults without dementia and 62% of patients with vascular dementia [1,4]. With advances in high-resolution magnetic resonance imaging (MRI), CCMI can now be detected in vivo [2,3,5]. Importantly, MRI-visible CCMI represents only a small fraction of the total microinfarct burden in the brain; accordingly, the detection of CCMI on neuroimaging may indicate a more widespread state of microvascular injury [3].

Beyond cognition, motor impairment is also a common and clinically important manifestation of CSVD, contributing to falls, disability, and loss of independence [6,7]. Growing evidence has linked CSVD burden to gait and other motor impairments [6,7,8]. However, the relationship between CCMI and motor function remains insufficiently understood. Existing evidence suggests that the impact of CCMI may extend beyond the small infarct core itself, involving perilesional tissue injury and disruption of remote brain networks [3,8]. These mechanisms may be particularly relevant to motor control, as gait, balance, lower-limb function, and complex gait regulation depend on distributed cortico-subcortical networks rather than on the primary motor cortex alone [9].

A clinicopathological study reported that microinfarcts were associated with late-life motor impairment [10], and a recent in vivo MRI study found poorer motor performance among individuals with cerebral microinfarcts [11]. However, previous evidence has been limited mainly by postmortem assessments or cross-sectional designs. Data are particularly scarce in patients with established CSVD, and whether CCMI is associated with baseline motor function and subsequent motor performance remains unclear. Clarifying these associations may help define the clinical significance of CCMI as an imaging marker of CSVD and determine its relevance to future motor outcomes.

On this basis, we hypothesized that CCMI would be associated with poorer motor function in patients with CSVD. In this secondary analysis of CIBB-CSVD data, we aimed to investigate the associations between CCMI and motor function at baseline and 1-year follow-up in patients with CSVD.

## 2. Materials and Methods

### 2.1. Study Design and Population

This secondary analysis used data collected at the Beijing Tiantan Hospital site of the China Imaging-based Biobank of Cerebral Small Vessel Diseases (CIBB-CSVD). CIBB-CSVD is an ongoing prospective observational cohort study designed to investigate imaging features, biomarkers, and clinical outcomes in patients with CSVD. Patients were eligible for CIBB-CSVD if they were aged ≥18 years, had a modified Rankin Scale (mRS) score ≤ 2, and met at least one of the following MRI criteria: white matter hyperintensity (WMH) Fazekas score ≥ 2; WMH Fazekas score = 1 with multiple vascular risk factors, including hypertension, hyperlipidemia, diabetes, obesity, and smoking; Fazekas score = 1 with lacune; or a recent subcortical lacunar infarct with an MRI diameter < 20 mm. Patients were excluded from CIBB-CSVD if they had acute large cerebral infarction, acute intracerebral or subarachnoid hemorrhage, diagnosed neurodegenerative disease, non-vascular white matter lesions, psychiatric disorders, contraindications to MRI, severe systemic disease, inability to comply with study procedures, or participation in other clinical trials. Between January 2020 and May 2023, 245 patients underwent baseline assessment at the Beijing Tiantan Hospital site of CIBB-CSVD. For this study, 192 patients with complete baseline brain MRI data and gait speed assessment were included in the baseline analysis. Among these patients, 74 had available 1-year follow-up gait speed data and were included in the longitudinal motor outcome analyses (Figure S1).

This study was performed in accordance with the principles of the Declaration of Helsinki. The CIBB-CSVD study was approved by the Ethics Committee of Beijing Tiantan Hospital (KY 2019-140-02), and written informed consent was obtained from all participants. The present analysis was reported following the STROBE guidelines for observational studies [12].

### 2.2. Clinical Assessment

Baseline variables included age, sex, education level, body-mass index, heart rate, height, history of hypertension, diabetes mellitus, hyperlipidemia, ever smoking, ever drinking, and Montreal Cognitive Assessment (MoCA) score.

### 2.3. Imaging Markers of CSVD

Patients with imaging findings suggestive of CSVD on prior clinical or screening MRI were screened for eligibility. After enrollment in CIBB-CSVD, all patients underwent an additional baseline brain MRI at Beijing Tiantan Hospital to assess CSVD imaging markers. MRI was performed using a 3T MRI scanner (MAGNETOM Prisma; Siemens, Erlangen, Germany) equipped with a 64-channel head coil. The standardized acquisition protocol included T1-weighted, T2-weighted, fluid-attenuated inversion recovery (FLAIR), diffusion-weighted imaging (DWI), and susceptibility-weighted imaging sequences. Detailed acquisition parameters and the primary purpose of each sequence are provided in Table S1.

**Figure 1:**
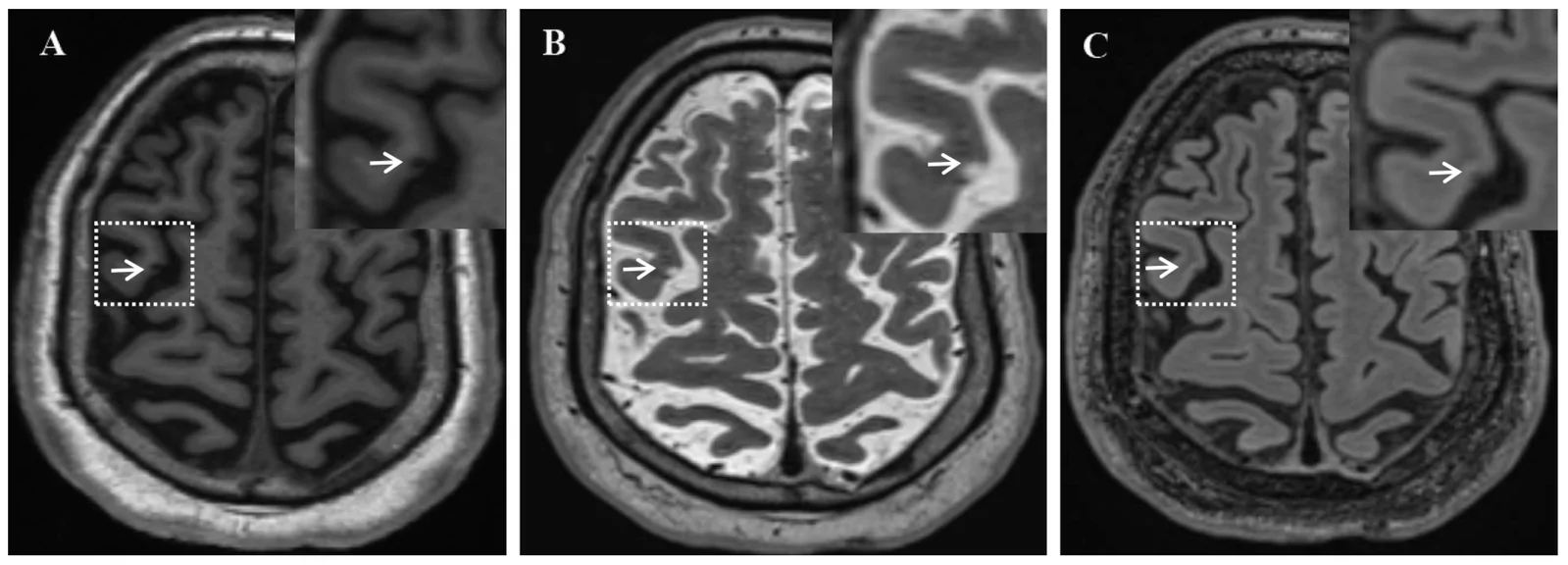
Example of a Cortical Cerebral Microinfarct on 3T MRI. White arrows indicate a cortical cerebral microinfarct appearing hypointense on T1-weighted imaging (**A**), hyperintense on T2-weighted imaging (**B**), and isointense on fluid-attenuated inversion recovery imaging relative to surrounding tissue (**C**). MRI: magnetic resonance imaging.

CSVD imaging markers were assessed according to the Standards for Reporting Vascular Changes on Neuroimaging 2 (STRIVE-2) [2]. Specifically, CCMI was defined according to established MRI criteria as a small lesion confined to the cortical gray matter, showing (i) hypointensity on T1-weighted images, (ii) hyperintensity on T2-weighted images, (iii) hyperintensity or isointensity on FLAIR images, (iv) diameter < 4 mm, and (v) an orientation perpendicular to the cortical surface (Figure 1). CCMI was independently rated by two trained neurologists (D.Z. and Le.G.), who were blinded to clinical data. Disagreements were adjudicated by a senior neurologist (T.W.). Inter-rater agreement for patient-level CCMI assessment was good (Cohen’s κ = 0.84).

Other CSVD imaging markers were also assessed. In brief, WMH was rated using the Fazekas scale [13], with a total score ranging from 0 to 6, including deep WMH (DWMH) and periventricular WMH (PVWMH). The presence of lacune was recorded. Cerebral microbleeds (CMBs) were counted, and ≥5 were defined as a moderate-to-severe burden. Enlarged perivascular space (EPVS) was assessed using a previously validated semi-quantitative scale, with grades ranging from 0 to 4, in the basal ganglia (BG-EPVS) and centrum semiovale (CSO-EPVS) [14]. Brain atrophy was evaluated using the global cortical atrophy scale; a score ≥ 2 was classified as moderate-to-severe brain atrophy [15]. The total CSVD burden score was calculated based on WMH, lacune, EPVS, and CMB, with a score ranging from 0 to 4; a score ≥ 2 was considered to indicate moderate-to-severe total CSVD burden. One point was assigned for each of the following: moderate-to-severe WMH (DWMH Fazekas score ≥ 2 or PVWMH Fazekas score = 3), presence of lacune, presence of CMB, and moderate-to-severe BG-EPVS (number > 10) [16].

### 2.4. Motor Function Assessment

Motor function was assessed face-to-face by uniformly trained neurologists at baseline and at the 1-year follow-up according to a standardized administration protocol (see Supplementary Method S1 for detailed procedures). In brief, gait speed was assessed on an 8 m level walkway, with the central 6 m timed and 1 m acceleration and deceleration zones at either end. Participants were instructed to walk independently at their usual pace. After one practice trial, three timed trials were performed, and gait speed was calculated from the mean completion time. Standing balance was assessed using the balance component of the Short Physical Performance Battery (SPPB), including side-by-side, semi-tandem, and tandem stands, each maintained for up to 10 s. Scores ranged from 0 to 4, with higher scores indicating better balance [17]. Consistent with prior work, an SPPB balance score ≤ 2 was categorized as poor balance performance [18]. Complex gait control was assessed using the gait item of the Scale for the Assessment and Rating of Ataxia (SARA), which included walking, turning 180°, and tandem walking. Scores ranged from 0 to 8, with higher scores indicating more severe gait abnormalities. A score of 2 indicates clearly abnormal gait; accordingly, scores ≥ 2 were categorized as abnormal gait in the present study [19]. Lower-limb function was assessed using the repeated chair-stand test on an armless chair, with participants’ arms crossed over the chest. Completion time was recorded when the participant reached the fully standing position after the fifth repetition, with longer completion time indicating poorer performance [17].

When planning this secondary analysis, gait speed was designated as the primary motor outcome, whereas poor balance performance, abnormal gait, and repeated chair-stand completion time were designated as secondary motor outcomes.

### 2.5. Statistical Analysis

Baseline characteristics were compared between participants with and without CCMI and between participants with and without 1-year follow-up assessments. Motor outcomes at the 1-year follow-up and their changes from baseline were also compared between participants with and without CCMI. Changes were calculated as the 1-year value minus the corresponding baseline value. Continuous variables were presented as mean ± standard deviation (SD) or median with interquartile range (IQR), as appropriate, and were compared using the t test or Wilcoxon rank-sum test according to their distribution. Categorical variables were presented as frequencies and percentages and were compared using the χ^2^ test or Fisher’s exact test, as appropriate.

CCMI status was treated as the exposure of interest in all regression analyses, with participants without CCMI serving as the reference group. Associations between CCMI and other CSVD imaging markers were examined using binary logistic regression or Firth’s penalized logistic regression, as appropriate. Models were adjusted for age, sex, and vascular risk factors (including hypertension, diabetes, hyperlipidemia, ever smoking, and ever drinking).

The primary regression analyses were based on complete cases. Gait speed and repeated chair-stand completion time were analyzed using linear regression, whereas poor balance performance and abnormal gait were analyzed using binary logistic regression or Firth’s penalized logistic regression, as appropriate. Results are reported as β coefficients for continuous outcomes and odds ratios (ORs) for binary outcomes, with corresponding 95% confidence intervals (CIs). Models for baseline motor outcomes were adjusted for age, sex, height, and vascular risk factors and total CSVD burden. Given the limited longitudinal sample size, the primary adjusted model for 1-year outcomes included age, sex, height, total CSVD burden and the corresponding baseline motor outcome.

To assess the robustness of the findings, two sensitivity analyses were performed. First, the analyses were repeated after excluding participants with any DWI-hyperintense lesion on baseline MRI, irrespective of lesion location, to evaluate the potential influence of recent ischemic lesions on motor function. Second, multiple imputation was performed to assess the potential influence of missing 1-year outcome data (see Supplementary Method S2 for details).

To account for multiple comparisons, the Benjamini–Hochberg (BH) correction was applied to the secondary motor outcomes in the baseline and 1-year analyses [20]. Both nominal and BH-adjusted *p* values were reported, with statistical significance determined by a BH-adjusted *p* value < 0.05. For the primary motor outcome, a two-sided *p* value < 0.05 was considered statistically significant. All statistical analyses were performed using SAS version 9.4 (SAS Institute, Cary, NC, USA).

## 3. Results

### 3.1. Baseline Characteristics

Among the 192 patients included in the baseline analysis, the mean age was 60.29 ± 11.21 years, and 76 patients (39.58%) were female. CCMI was present in 55 patients (28.65%) and absent in 137 patients (71.35%). Among the 86 CCMI lesions identified in these 55 patients, 44 (51.16%) were located in the frontal lobe. Further details on CCMI burden and anatomical distribution are provided in Table S2. Compared with patients without CCMI, those with CCMI were older (63.07 ± 8.74 years vs. 59.18 ± 11.91 years, *p* = 0.03) and had lower education-adjusted MoCA scores (18.00 [13.50–21.50] vs. 21.00 [17.00–25.00], *p* = 0.002). No significant differences were observed in other baseline demographic characteristics or vascular risk factors between the two groups (all *p* > 0.05; Table 1). Among the 74/192 (38.54%) patients with available 1-year motor follow-up data, 26 (35.14%) had CCMI and 48 (64.86%) did not. Compared with patients without 1-year follow-up, those with follow-up had higher education-adjusted MoCA scores (22.00 [18.00–24.00] vs. 19.00 [15.00–24.00], *p* = 0.007) and shorter repeated chair-stand time at baseline (11.52 [9.39–13.43] s vs. 12.21 [9.85–16.07] s, *p* = 0.04), whereas no significant differences were observed in demographic characteristics, vascular risk factors, other motor function measures, or CSVD imaging markers (Table S3).

### 3.2. Association Between CCMI and Other CSVD Imaging Markers

In the comparison of CSVD imaging markers between groups, patients with CCMI showed a higher overall CSVD imaging burden. Compared with patients without CCMI, those with CCMI had a higher proportion of moderate-to-severe total CSVD burden (92.73% vs. 67.15%, *p* < 0.001). They also had higher prevalences of moderate-to-severe PVWMH (90.91% vs. 73.72%), DWMH (89.09% vs. 67.15%), BG-EPVS (78.18% vs. 59.12%), CMB (45.45% vs. 25.55%), brain atrophy (58.18% vs. 42.34%) and presence of lacunes (89.09% vs. 60.58%) (all *p* < 0.05; Table 1).

Logistic regression was further used to examine the association between CCMI and other CSVD imaging markers. After adjustment for age, sex, and vascular risk factors, CCMI remained significantly associated with moderate-to-severe total CSVD burden (adjusted OR 4.65, 95% CI: 1.65–13.09, *p* = 0.004). For individual imaging markers, CCMI was associated with moderate-to-severe PVWMH (adjusted OR 2.88, 95% CI: 1.10–7.54, *p* = 0.03), moderate-to-severe DWMH (adjusted OR 3.33, 95% CI: 1.35–8.24, *p* = 0.009), lacune (adjusted OR 4.67, 95% CI: 1.87–11.64, *p* < 0.001), and moderate-to-severe CMB (adjusted OR 2.42, 95% CI: 1.21–4.85, *p* = 0.01). No statistically significant associations were observed for moderate-to-severe BG-EPVS, CSO-EPVS, or brain atrophy (Table 2).

### 3.3. Association Between CCMI and Baseline Motor Function

At baseline, patients with CCMI showed poorer performance in balance and gait-related measures. Compared with patients without CCMI, those with CCMI had lower gait speed [0.92 (0.75–1.12) m/s vs. 1.05 (0.87–1.16) m/s], a higher prevalence of poor balance performance (29.09% vs. 14.60%), and abnormal gait (63.64% vs. 36.50%) (all *p* < 0.05). Repeated chair-stand time did not differ significantly between the groups [12.24 (10.30–16.74) s vs. 11.76 (9.25–14.93) s, *p* = 0.09; Table 1].

After adjustment for age, sex, height, and vascular risk factors, CCMI was significantly associated with poor balance performance (adjusted OR 3.16, 95% CI: 1.34–7.47, *p* = 0.01) and abnormal gait (adjusted OR 3.27, 95% CI: 1.59–6.72, *p* = 0.001). No significant associations were observed with gait speed (adjusted β −0.07, 95% CI: −0.15 to 0.02, *p* = 0.12) or repeated chair-stand time (adjusted β 0.82, 95% CI: −0.72 to 2.36, *p* = 0.30).

After additional adjustment for total CSVD burden, the association with poor balance performance was attenuated and no longer statistically significant (adjusted OR 2.18, 95% CI: 0.88–5.43, *p* = 0.09, adjusted *p* = 0.14). CCMI remained nominally associated with abnormal gait, but did not survive BH correction (adjusted OR 2.35, 95% CI: 1.10–5.02, *p* = 0.03, adjusted *p* = 0.09; Table 3).

### 3.4. Association Between CCMI and 1-Year Follow-Up Motor Function

At the 1-year follow-up, compared with patients without CCMI, those with CCMI had lower gait speed [0.93 (0.80–1.03) m/s vs. 1.11 (1.00–1.29) m/s, *p* < 0.001], and longer repeated chair-stand time [13.61 (10.46–16.21) s vs. 11.00 (8.50–12.72) s, *p* = 0.02; Table S4]. Poor balance performance was more frequent among patients with CCMI, although the difference did not reach statistical significance (23.08% vs. 6.25%, *p* = 0.06), whereas abnormal gait was significantly more frequent (57.69% vs. 25.00%, *p* = 0.005; score distributions are presented in Table S5).

From baseline to 1 year, the median change in gait speed differed between patients with and without CCMI [−0.04 (−0.19 to 0.05) m/s vs. 0.08 (−0.02 to 0.16) m/s, *p* = 0.002]. Patients with CCMI also more frequently exhibited gait worsening (30.77% vs. 4.17%, *p* = 0.003) and had a greater increase in repeated chair-stand time [0.75 (−1.02 to 3.25) s vs. 0.26 (−1.68 to 0.69) s, *p* = 0.04; Table S4].

After adjustment for age, sex, height, total CSVD burden, and the corresponding baseline motor performance, CCMI was associated with slower gait speed (adjusted β −0.16, 95% CI: −0.24 to −0.08, *p* < 0.001). The association with longer repeated chair-stand time did not survive BH correction (adjusted β 2.07, 95% CI: 0.39–3.75, *p* = 0.02, adjusted *p* = 0.06). No significant associations were observed with abnormal gait (adjusted OR 2.11, 95% CI: 0.57–7.82, *p* = 0.26, adjusted *p* = 0.26) or poor balance performance (adjusted OR 4.17, 95% CI: 0.72–24.13, *p* = 0.11, adjusted *p* = 0.17) (Table 4).

In sensitivity analyses, a consistent association between CCMI and slower gait speed was observed after excluding patients with any DWI-hyperintense lesion on baseline MRI (adjusted β −0.16, 95% CI: −0.24 to −0.08, *p* < 0.001; Table S6). The results following multiple imputation for missing 1-year gait speed data were also consistent with the main analysis (adjusted β −0.16, 95% CI: −0.24 to −0.08, *p* < 0.001; Tables S7 and S8).

## 4. Discussion

In this secondary analysis of CIBB-CSVD data, we found that CCMI was associated with a higher burden of conventional CSVD markers, including WMH, lacunes, and CMBs. Patients with CCMI showed poorer baseline motor performance, and CCMI was associated with poor balance performance and abnormal gait after adjustment for demographic and vascular risk factors; however, the effect estimates were attenuated after further adjustment for total CSVD burden, and neither association survived correction for multiple comparisons. At the 1-year follow-up, CCMI was associated with slower gait speed, even after adjustment for total CSVD burden and corresponding baseline motor performance. Although the point estimates for abnormal gait and chair-stand time were also in the direction of poorer performance, these effect estimates were imprecise, likely in part because of the limited longitudinal sample (n = 74), and neither secondary association provided conclusive evidence after correction for multiple comparisons. Thus, the present findings raise the possibility that baseline CCMI is associated with slower gait speed at the 1-year follow-up.

Our findings are consistent with previous studies showing that motor impairment is an important clinical manifestation of CSVD [7]. Prior studies have shown that conventional CSVD markers, including WMH, lacunes, and CMBs, are associated with gait disturbance and impaired balance [6,21,22,23]. However, evidence regarding the role of CCMI in motor dysfunction remains limited, particularly in longitudinal settings. A previous cross-sectional study of dementia-free participants found that those with DWI-positive cerebral microinfarcts had poorer balance and chair-stand performance, but did not differ in gait speed [11]. Multiple factors may account for the differences in motor phenotypes between that study and ours, including heterogeneity between the study populations and random variation due to the small sample sizes.

Previous studies have suggested that CSVD imaging markers may be interrelated and can evolve dynamically over time [2,24]. CCMI and other CSVD markers may share common upstream pathology, such as arteriolosclerosis [3,25]. At the same time, CCMI may induce tissue responses that extend beyond the visible lesion core, including neuroinflammation, reactive gliosis, and microstructural disruption, which could potentially influence the progression or clinical expression of CSVD lesions [1,3,26]. In our study, the attenuation of the association between CCMI and baseline motor performance after adjustment for overall CSVD burden suggests that the relationship between CCMI and motor performance may be embedded in a broader CSVD pathological process, rather than reflecting the isolated effect of CCMI alone. WMH may impair motor function by disrupting frontal–subcortical circuits and long-range white matter pathways [7], whereas lacunes and CMBs in motor-related subcortical regions, particularly the basal ganglia or brainstem, may affect posture and gait regulation [22,23,27,28]. When CCMI coexists with these lesions, it may further reduce the redundancy and compensatory capacity of motor networks. Importantly, MRI-visible CCMIs likely represent only a small fraction of the total cerebral microinfarct burden, and recent longitudinal MRI studies have also shown that the visibility of CCMIs may change over time [29,30,31]. Their presence may therefore indicate a much greater underlying microinfarct burden than is apparent from the few lesions detected on MRI [1,3,4].

For the primary motor outcome of gait speed, patients with CCMI had slower gait speed at both baseline and 1-year follow-up. The adjusted estimates were directionally consistent across time points, although evidence for an adjusted association was more robust at the 1-year follow-up. The reasons for this difference remain unclear. More generally, several mechanisms may link CCMI to poorer motor performance. Experimental studies in rodents suggest that microinfarcts may induce persistent tissue injury extending beyond the infarct core [32]. Neuroimaging studies have also reported perilesional and remote effects, including perilesional cortical atrophy, impaired functional connectivity, and network-level reorganization [11,30,33,34]. Because gait and other motor functions depend on widely distributed cortical-subcortical networks, we speculate that persistent microinfarct-related tissue injury and impaired functional connectivity may disrupt these networks and contribute to poorer subsequent motor performance in patients with CCMI [35]. However, these potential mechanisms remain speculative and require further validation in future studies.

The potential role of lesion location should also be considered. Among the 86 CCMI lesions identified in our study, 44 (51.16%) were located in the frontal lobe (Table S2). Frontal cortical-subcortical networks are important for gait control, and frontal lacunes have been associated with slower gait in patients with CSVD [9,27]. Further studies are needed to examine location-specific associations between CCMI and gait speed.

Cognitive impairment may also contribute to the association between CCMI and gait performance. CCMI has been associated with cognitive impairment [1,3], while executive function and attention are important for gait control [6]. Thus, cognition may partly mediate this association. However, the modest sample size limited our ability to conduct a robust mediation analysis, and this possibility should be evaluated in larger longitudinal cohorts.

Among the secondary motor outcomes, CCMI was nominally associated with abnormal gait at baseline and longer repeated chair-stand time at the 1-year follow-up. However, neither association survived correction for multiple comparisons; therefore, these findings should be considered hypothesis-generating and interpreted cautiously.

Several limitations should be acknowledged. First, 1-year motor assessments were unavailable for 118 of 192 patients (61.46%), primarily because 113 patients could not complete face-to-face assessments owing to COVID-19-related restrictions. Although results were consistent in the multiple-imputation analysis, bias arising from missing follow-up outcome data cannot be excluded. Second, the small longitudinal sample may have led to overestimated effect sizes and limited further analyses by CCMI number or location. In particular, the relationship between the distribution of multiple lesions within the same patient and motor outcomes was not examined. The single-center, hospital-based setting may also limit generalizability. Larger multicenter longitudinal studies are needed to confirm our findings. Third, MRI may underestimate the true burden of CCMI because of its limited spatial resolution compared with pathological examination. Although CCMI was independently rated by two experienced neurologists with good inter-rater agreement in this study, future studies may explore deep learning-assisted or automated imaging methods to improve detection sensitivity and reproducibility of CCMI. Fourth, although this study used clinically applicable motor tests, refined quantitative gait assessment was not performed. Future studies using quantitative gait analysis may help more comprehensively characterize the motor phenotype associated with CCMI. Finally, information on treatments, rehabilitation, physical activity, and major health events during follow-up was not systematically collected. As a result, these factors could not be accounted for in the analysis, and residual confounding by these or other clinical factors cannot be excluded.

## 5. Conclusions

In conclusion, our study suggests that CCMI is associated with slower gait speed at 1-year follow-up in patients with CSVD. Given the limited longitudinal sample, this finding should be considered hypothesis-generating and requires confirmation in larger longitudinal studies.

## Figures and Tables

**Table 1:** Baseline Characteristics of Patients According to CCMI Status.

	With CCMI (n = 55)	Without CCMI (n = 137)	*p* Value
Age ^a^, years	63.07 (8.74)	59.18 (11.91)	0.03
Female	25 (45.45)	51 (37.23)	0.29
Education level ^b^	48 (87.27)	119 (86.86)	0.94
Education-adjusted MoCA score, points	18.00 (13.50–21.50)	21.00 (17.00–25.00)	0.002
Body-mass index, kg/m^2^	24.91 (23.05–27.04)	25.39 (23.88–27.73)	0.28
Heart rate, beats/min	72.00 (66.00–78.00)	72.00 (68.00–78.00)	0.29
Height ^a^, cm	164.89 (9.98)	166.29 (7.73)	0.30
Medical history			
Hypertension	36 (65.45)	88 (64.23)	0.87
Diabetes	11 (20.00)	39 (28.47)	0.23
Hyperlipidemia	17 (30.91)	27 (19.71)	0.10
Ever smoking	23 (41.82)	62 (45.26)	0.66
Ever drinking	23 (41.82)	64 (46.72)	0.54
Motor function			
Gait speed, m/s	0.92 (0.75–1.12)	1.05 (0.87–1.16)	0.02
Poor balance ^c^	16 (29.09)	20 (14.60)	0.02
Abnormal gait ^d^	35 (63.64)	50 (36.50)	<0.001
Repeated chair-stand time, s	12.24 (10.30–16.74)	11.76 (9.25–14.93)	0.09
CSVD imaging markers			
Total CSVD burden ^e^	51 (92.73)	92 (67.15)	<0.001
Periventricular WMH ^e^	50 (90.91)	101 (73.72)	0.01
Deep WMH ^e^	49 (89.09)	92 (67.15)	0.002
BG-EPVS ^e^	43 (78.18)	81 (59.12)	0.01
CSO-EPVS ^e^	31 (56.36)	89 (64.96)	0.27
Lacune	49 (89.09)	83 (60.58)	<0.001
CMB ^e^	25 (45.45)	35 (25.55)	0.007
Brain atrophy ^e^	32 (58.18)	58 (42.34)	0.047

Data are n (%) or median (interquartile range) unless otherwise indicated. MoCA scores were adjusted for education by adding 1 point for participants with 12 years of education or fewer. ^a^ Data are mean (standard deviation). ^b^ Primary school or above. ^c^ A score ≤ 2 on the balance component (0–4, with higher scores indicating better balance) of the Short Physical Performance Battery. ^d^ A score ≥ 2 on the gait item (0–8, with higher scores indicating greater gait impairment) of the Scale for the Assessment and Rating of Ataxia. ^e^ Moderate-to-severe. CCMI: cortical cerebral microinfarct; MoCA: Montreal Cognitive Assessment; CSVD: cerebral small vessel disease; WMH: white matter hyperintensities; EPVS: enlarged perivascular spaces; BG: basal ganglia; CSO: centrum semiovale; CMB: cerebral microbleed.

**Table 2:** Associations Between CCMI and Other CSVD Imaging Markers.

	Unadjusted		Model 1		Model 2	
	OR (95% CI)	*p* Value	OR (95% CI)	*p* Value	OR (95% CI)	*p* Value
Total CSVD burden ^a,b^	5.63 (2.00–15.84)	0.001	4.88 (1.71–13.90)	0.003	4.65 (1.65–13.09)	0.004
Periventricular WMH ^a,b^	3.30 (1.26–8.68)	0.02	3.06 (1.16–8.06)	0.02	2.88 (1.10–7.54)	0.03
Deep WMH ^a,b^	3.75 (1.53–9.20)	0.004	3.67 (1.49–9.07)	0.005	3.33 (1.35–8.24)	0.009
BG-EPVS ^a^	2.48 (1.20–5.11)	0.01	2.07 (0.97–4.44)	0.06	2.06 (0.93–4.59)	0.08
CSO-EPVS ^a^	0.70 (0.37–1.32)	0.27	0.58 (0.30–1.13)	0.11	0.59 (0.30–1.17)	0.13
Lacune ^b^	4.97 (2.04–12.15)	<0.001	4.73 (1.91–11.71)	<0.001	4.67 (1.87–11.64)	<0.001
CMB ^a^	2.43 (1.26–4.68)	0.008	2.40 (1.23–4.66)	0.01	2.42 (1.21–4.85)	0.01
Brain atrophy ^a^	1.90 (1.01–3.57)	0.048	1.78 (0.90–3.53)	0.10	1.99 (0.98–4.06)	0.06

Patients without CCMI were used as the reference group in all models. Patients with CCMI, n = 55; patients without CCMI, n = 137. Model 1: Adjusted for age, sex. Model 2: Adjusted for age, sex, hypertension, diabetes, hyperlipidemia, ever smoking, and ever drinking. ^a^ Moderate-to-severe. ^b^ Estimated using Firth’s penalized logistic regression because of sparse data. CCMI: cortical cerebral microinfarct; CSVD: cerebral small vessel disease; OR: odds ratio; CI: confidence interval; WMH: white matter hyperintensities; EPVS: enlarged perivascular spaces; BG: basal ganglia; CSO: centrum semiovale; CMB: cerebral microbleed.

**Table 3:** Associations Between CCMI and Motor Function at Baseline.

	Unadjusted		Model A		Model B		
	β/OR (95% CI)	*p* Value	β/OR (95% CI)	*p* Value	β/OR (95% CI)	*p* Value	BH-Adjusted *p* Value
Baseline Motor Function ^a^							
Gait speed	−0.09 (−0.18–−0.01)	0.03	−0.07 (−0.15–0.02)	0.12	−0.03 (−0.11–0.06)	0.56	—
Poor balance ^b^	2.40 (1.13–5.08)	0.02	3.16 (1.34–7.47)	0.01	2.18 (0.88–5.43)	0.09	0.14
Abnormal gait ^c^	3.05 (1.59–5.83)	<0.001	3.27 (1.59–6.72)	0.001	2.35 (1.10–5.02)	0.03	0.09
Repeated chair-stand time	1.16 (−0.43–2.76)	0.16	0.82 (−0.72–2.36)	0.30	0.09 (−1.49–1.67)	0.91	0.91

Patients without CCMI were used as the reference group in all models. Gait speed was designated as the primary motor outcome and was therefore not included in the BH adjustment. BH-adjusted *p* values were calculated from the nominal *p* values in Model B. Model A: Adjusted for age, sex, height, hypertension, diabetes, hyperlipidemia, ever smoking, and ever drinking. Model B: Adjusted for age, sex, height, hypertension, diabetes, hyperlipidemia, ever smoking, ever drinking, and total CSVD burden. ^a^ Patients with CCMI, n = 55; patients without CCMI, n = 137. ^b^ A score ≤ 2 on the balance component of the Short Physical Performance Battery. Events occurred in 16/55 patients with CCMI and 20/137 patients without CCMI. ^c^ A score ≥ 2 on the gait item of the Scale for the Assessment and Rating of Ataxia. Events occurred in 35/55 patients with CCMI and 50/137 patients without CCMI. CI: confidence interval; OR: odds ratio; BH: Benjamini–Hochberg; CCMI: cortical cerebral microinfarct; CSVD: cerebral small vessel disease.

**Table 4:** Associations Between CCMI and Motor Function at 1-Year Follow-Up.

	Unadjusted		Model C		Model D		
	β/OR (95% CI)	*p* Value	β/OR (95% CI)	*p* Value	β/OR (95% CI)	*p* Value	BH-Adjusted *p* Value
1-Year Follow-Up Motor Function ^a^							
Gait speed	−0.22 (−0.33–−0.12)	<0.001	−0.17 (−0.25–−0.09)	<0.001	−0.16 (−0.24–−0.08)	<0.001	—
Poor balance ^b,d^	4.12 (0.997–17.04)	0.051	4.18 (0.82–21.30)	0.09	4.17 (0.72–24.13)	0.11	0.17
Abnormal gait ^c^	4.09 (1.48–11.30)	0.007	2.31 (0.69–7.77)	0.17	2.11 (0.57–7.82)	0.26	0.26
Repeated chair-stand time	3.48 (1.18–5.79)	0.004	2.01 (0.36–3.66)	0.02	2.07 (0.39–3.75)	0.02	0.06

Patients without CCMI were used as the reference group in all models. Gait speed was designated as the primary motor outcome and was therefore not included in the BH adjustment. BH-adjusted *p* values were calculated from the nominal *p* values in Model D. Model C: Adjusted for age, sex, height, and the corresponding baseline motor outcome. Model D: Adjusted for age, sex, height, total CSVD burden, and the corresponding baseline motor outcome. ^a^ Patients with CCMI, n = 26; patients without CCMI, n = 48. ^b^ A score ≤ 2 on the balance component of the Short Physical Performance Battery. Events occurred in 6/26 patients with CCMI and 3/48 patients without CCMI. ^c^ A score ≥ 2 on the gait item of the Scale for the Assessment and Rating of Ataxia. Events occurred in 15/26 patients with CCMI and 12/48 patients without CCMI. ^d^ Estimated using Firth’s penalized logistic regression because of sparse data. CI: confidence interval; OR: odds ratio; BH: Benjamini–Hochberg; CCMI: cortical cerebral microinfarct; CSVD: cerebral small vessel disease.

## Data Availability

The data that support the findings of this study are available from the corresponding author on reasonable request. Data are not publicly available due to privacy reasons.

## Supplementary Materials

The following supporting information can be downloaded at: https://www.mdpi.com/article/10.3390/brainsci16080892/s1, Figure S1. Flow Chart. Table S1. Detailed MRI scan parameters and sequence purposes in the present study. Table S2. Baseline CCMI lesion burden and distribution. Table S3. Baseline Characteristics of Patients With and Without 1-Year Follow-Up Assessment. Table S4. One-Year Motor Function and Changes From Baseline by CCMI Status. Table S5. Distribution of SPPB Balance and SARA Gait Scores at Baseline and 1-Year Follow-Up According to Baseline CCMI Status. Table S6. Sensitivity Analysis of the Association Between CCMI and Gait Speed at the 1-Year Follow-Up (excluding participants with baseline DWI-hyperintense lesions). Table S7. Associations Between CCMI and Motor Function at 1-Year Follow-Up (Multiple Imputation). Table S8. Estimates of the Association Between CCMI and Gait Speed at 1-Year Follow-Up in the Primary and Sensitivity Analyses. Supplementary Method S1. Motor and Gait Assessment Protocol. Supplementary Method S2. Multiple imputation of missing 1-year motor outcome data.

## Abbreviations

The following abbreviations are used in this manuscript:

BG	basal ganglia
BG-EPVS	basal ganglia enlarged perivascular space
BH	Benjamini–Hochberg
CI	confidence interval
CIBB-CSVD	China Imaging-based Biobank of Cerebral Small Vessel Diseases
CMB	cerebral microbleed
CCMI	cortical cerebral microinfarct
COVID-19	coronavirus disease 2019
CSO	centrum semiovale
CSO-EPVS	centrum semiovale enlarged perivascular space
CSVD	cerebral small vessel disease
DWI	diffusion-weighted imaging
DWMH	deep white matter hyperintensity
EPVS	enlarged perivascular space
FLAIR	fluid-attenuated inversion recovery
IQR	interquartile range
MoCA	Montreal Cognitive Assessment
MRI	magnetic resonance imaging
mRS	modified Rankin Scale
OR	odds ratio
PVWMH	periventricular white matter hyperintensity
SARA	Scale for the Assessment and Rating of Ataxia
SAS	Statistical Analysis System
SD	standard deviation
SPPB	Short Physical Performance Battery
STRIVE-2	Standards for Reporting Vascular Changes on Neuroimaging 2
STROBE	Strengthening the Reporting of Observational Studies in Epidemiology
WMH	white matter hyperintensity
