# Supplementary material for: Association Between Cortical Cerebral Microinfarct and Motor Performance at One-Year Follow-Up in Patients with Cerebral Small Vessel Disease: An Exploratory Study
Source: Brain Sci. 2026 Aug 20;16(8):892. doi: 10.3390/brainsci16080892 (PMC13510354; doi:10.3390/brainsci16080892)
Supplement: Supplementary file 1 [file brainsci-16-00892-s001.zip › brainsci-4464509-supplementary.pdf]

Supplementary Materials

Association Between Cortical Cerebral Microinfarct and Motor Performance at One-Year Follow-Up in Patients with Cerebral Small Vessel Disease: An Exploratory Study

**Table of Contents**	**Page**
Figure S1. Flow chart	2
Table S1. Detailed MRI scan parameters and sequence purposes in the present study	3
Table S2. Baseline CCMI lesion burden and distribution	4
Table S3. Baseline characteristics of patients with and without 1-year follow-up assessment	5
Table S4. One-year motor function and changes from baseline by CCMI status	6
Table S5. Distribution of SPPB balance and SARA gait scores at baseline and 1-year follow-up according to baseline CCMI status	7
Table S6. Sensitivity analysis of the association between CCMI and gait speed at the 1-year follow-up (excluding participants with baseline DWI-hyperintense lesions)	8
Table S7. Associations between CCMI and motor function at 1-year follow-up (multiple imputation)	8
Table S8. Estimates of the association between CCMI and gait speed at 1-year follow-up in the primary and sensitivity analyses	9
Supplementary Method S1. Motor and Gait Assessment Protocol	10
Supplementary Method S2. Multiple imputation of missing 1-year motor outcome data	13

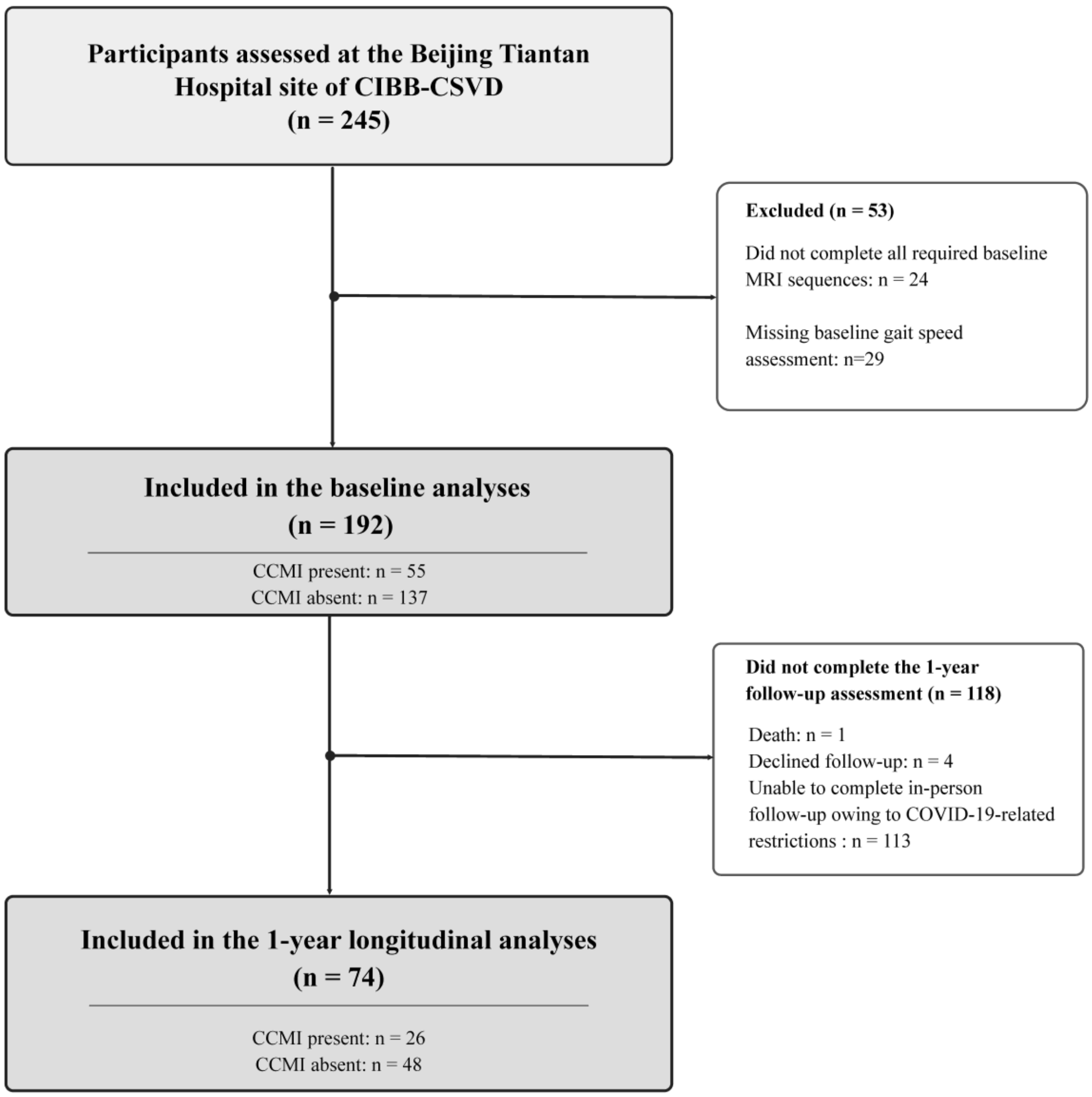

Figure S1. Flow chart

CIBB-CSVD, China Imaging-based Biobank of Cerebral Small Vessel Diseases; CCMI, cortical cerebral microinfarct; MRI, magnetic resonance imaging.

Table S1. Detailed MRI scan parameters and sequence purposes in the present study

Contrast	TR/ms	TI/ms	TE/ms	FOV/mm³	Voxel Size /mm³	Slice Thickness /mm	FA/°	*b*, s/mm²	Main purpose(s)
T1-weighted	2300	900	2.3	256×256 ×196	1.0×1.0 ×1.0	1.0	8	NA	Anatomical reference and assessment of CCMI and brain atrophy
T2-weighted	3000	NA	409	256×256 ×196	1.0×1.0 ×1.0	1.0	120	NA	Assessment of CCMI and EPVS
FLAIR	5000	1800	386	256×256 ×196	1.0×1.0 ×1.0	1.0	40	NA	Assessment of CCMI, WMH and lacunes
DWI	6100	NA	65	184×226 ×143	1.6×2.0 ×5.0	5.0	NA	1200	Assessment of recent infarcts
SWI	29	NA	20	210×240 ×159	0.5×0.5 ×1.5	1.5	15	NA	Assessment of CMBs

NA: not applicable; CCMI: cortical cerebral microinfarct; WMH: white matter hyperintensities; EPVS: enlarged perivascular spaces; CMB: cerebral microbleed; FLAIR: fluid-attenuated inversion recovery; DWI: diffusion-weighted imaging; SWI: susceptibility-weighted imaging; TR: repetition time; TI: inversion time; TE: echo time; FOV: field of view; FA: flip angle.

Table S2. Baseline CCMI lesion burden and distribution

Panel A. Participant-level lesion burden (n = 55)

Lesion burden	Participants, n (%)
Single CCMI lesion	25 (45.45)
Multiple CCMI lesions	30 (54.55)

Panel B. Lesion-level anatomical distribution (86 CCMI lesions)

Anatomical region	Left hemisphere, n (%)	Right hemisphere, n (%)	Total, n (%)
Frontal lobe	25 (29.07)	19 (22.09)	44 (51.16)
Parietal lobe	11 (12.79)	5 (5.81)	16 (18.60)
Temporal lobe	2 (2.33)	5 (5.81)	7 (8.14)
Occipital lobe	13 (15.12)	6 (6.98)	19 (22.09)
Total	51 (59.30)	35 (40.70)	86 (100.00)

Data are presented as n (%). Percentages may not sum to 100.00% because of rounding. Panel A percentages were calculated among participants with CCMI (n = 55); Panel B percentages were calculated using all CCMI lesions (n = 86). A single CCMI lesion indicates one lesion, and multiple CCMI lesions indicate two or more lesions. Each lesion was counted separately, so participants with multiple lesions could contribute to more than one anatomical category. CCMI: cortical cerebral microinfarct.

Table S3. Baseline characteristics of patients with and without 1-year follow-up assessment

	**With 1-year follow-up (n=74)**	**Without 1-year follow-up (n=118)**	***p* value**
Age[a], years	60.49 (9.65)	60.17 (12.12)	0.85
Female	33 (44.59)	43 (36.44)	0.26
Education level[b]	66 (89.19)	101 (85.59)	0.47
Education-adjusted MoCA score, points	22.00 (18.00-24.00)	19.00 (15.00-24.00)	0.007
Body-mass index, kg/m^2	25.39 (22.77-26.88)	25.21 (23.73-27.74)	0.07
Heart rate, beats/min	70.50 (68.00-78.00)	72.00 (68.00-78.00)	0.73
Height[a], cm	165.58 (9.06)	166.08 (8.04)	0.69
**Medical history**			
Hypertension	46 (62.16)	78 (66.10)	0.58
Diabetes	23 (31.08)	27 (22.88)	0.21
Hyperlipidemia	18 (24.32)	26 (22.03)	0.71
Ever smoking	30 (40.54)	55 (46.61)	0.41
Ever drinking	32 (43.24)	55 (46.61)	0.65
**Motor function**			
Gait speed, m/s	1.06 (0.90-1.17)	0.99 (0.76-1.14)	0.15
Poor balance[c]	15 (20.27)	21 (17.80)	0.67
Abnormal gait[d]	33 (44.59)	52 (44.07)	0.94
Repeated chair-stand time, s	11.52 (9.39-13.43)	12.21 (9.85-16.07)	0.04
**CSVD imaging markers**			
Total CSVD burden[e]	55 (74.32)	88 (74.58)	0.97
CCMI	26 (35.14)	29 (24.58)	0.12
Periventricular WMH[e]	58 (78.38)	93 (78.81)	0.94
Deep WMH[e]	55 (74.32)	86 (72.88)	0.83
BG-EPVS[e]	52 (70.27)	72 (61.02)	0.19
CSO-EPVS[e]	52 (70.27)	68 (57.63)	0.08
Lacune	51 (68.92)	81 (68.64)	0.97
CMB[e]	23 (31.08)	37 (31.36)	0.97
Brain atrophy[e]	35 (47.30)	55 (46.61)	0.93

Data are n (%) or median (interquartile range) unless otherwise indicated. MoCA scores were adjusted for education by adding 1 point for participants with 12 years of education or fewer.

[a]Data are mean (standard deviation).

[b]Primary school or above.

[c]A score ≤2 on the balance component (0–4, with higher scores indicating better balance) of the Short Physical Performance Battery.

[d]A score ≥2 on the gait item (0–8, with higher scores indicating greater gait impairment) of the Scale for the Assessment and Rating of Ataxia.

[e]Moderate-to-severe.

CCMI: cortical cerebral microinfarct; MoCA: Montreal Cognitive Assessment; CSVD: cerebral small vessel disease; WMH: white matter hyperintensities; EPVS: enlarged perivascular spaces; BG: basal ganglia; CSO: centrum semiovale; CMB: cerebral microbleed.

Table S4. One-year motor function and changes from baseline by CCMI status

Motor Function Outcome	Baseline CCMI Status With CCMI (n=26)	Baseline CCMI Status Without CCMI (n=48)	*p* value
**Motor Function at 1-Year Follow-Up**			
Gait speed, m/s	0.93 (0.80-1.03)	1.11 (1.00-1.29)	<0.001
Poor balance, n (%)	6 (23.08)	3 (6.25)	0.06
Abnormal gait, n (%)	15 (57.69)	12 (25.00)	0.005
Repeated chair-stand time, s	13.61 (10.46-16.21)	11.00 (8.50-12.72)	0.02
Changes From Baseline to 1-Year Follow-Up[a]			
Change in gait speed, m/s	-0.04 (-0.19-0.05)	0.08 (-0.02-0.16)	0.002
Improved standing balance, n (%)	7 (26.92)	8 (16.67)	0.29
Worsened standing balance, n (%)	2 (7.69)	1 (2.08)	0.28
Improved gait performance, n (%)	7 (26.92)	14 (29.17)	0.84
Worsened gait performance, n (%)	8 (30.77)	2 (4.17)	0.003
Change in repeated chair-stand time, s	0.75 (-1.02-3.25)	0.26 (-1.68-0.69)	0.04

Data are presented as n (%) or median (interquartile range).

[a]Changes were calculated as the value at the 1-year follow-up minus the baseline value. Improved standing balance was indicated by an increase in the SPPB balance score, whereas worsened standing balance was indicated by a decrease. Improved gait performance was indicated by a decrease in the SARA gait score, whereas worsened gait performance was indicated by an increase.

Table S5. Distribution of SPPB balance and SARA gait scores at baseline and 1-year follow-up according to baseline CCMI status

Baseline SPPB balance score

Baseline CCMI status	Score 0	Score 1	Score 2	Score 3	Score 4
With CCMI	2 (3.64)	2 (3.64)	12 (21.82)	15 (27.27)	24 (43.64)
Without CCMI	6 (4.38)	1 (0.73)	13 (9.49)	22 (16.06)	95 (69.34)

Baseline SARA gait score

Baseline CCMI status	Score 0	Score 1	Score 2	Score 3	Score 4	Score 5	Score 6	Score 7	Score 8
With CCMI	13 (23.64)	7 (12.73)	25 (45.45)	3 (5.45)	4 (7.27)	1 (1.82)	1 (1.82)	0 (0.00)	1 (1.82)
Without CCMI	63 (45.99)	24 (17.52)	38 (27.74)	3 (2.19)	6 (4.38)	2 (1.46)	1 (0.73)	0 (0.00)	0 (0.00)

1-year follow-up SPPB balance score

Baseline CCMI status	Score 0	Score 1	Score 2	Score 3	Score 4
With CCMI	1 (3.85)	0 (0.00)	5 (19.23)	5 (19.23)	15 (57.69)
Without CCMI	0 (0.00)	1 (2.08)	2 (4.17)	8 (16.67)	37 (77.08)

1-year follow-up SARA gait score

Baseline CCMI status	Score 0	Score 1	Score 2	Score 3	Score 4	Score 5	Score 6	Score 7	Score 8
With CCMI	7 (26.92)	4 (15.38)	12 (46.15)	0 (0.00)	0 (0.00)	1 (3.85)	1 (3.85)	0 (0.00)	1 (3.85)
Without CCMI	33 (68.75)	3 (6.25)	11 (22.92)	1 (2.08)	0 (0.00)	0 (0.00)	0 (0.00)	0 (0.00)	0 (0.00)

Data are presented as n (%), with percentages calculated within each baseline CCMI group. At baseline, patients with CCMI, n = 55; patients without CCMI, n = 137. At 1-year follow-up, patients with CCMI, n = 26; patients without CCMI, n = 48. The SPPB balance score ranges from 0 to 4, with higher scores indicating better standing balance. The SARA gait score ranges from 0 to 8, with higher scores indicating greater gait impairment.

SPPB: Short Physical Performance Battery; SARA: Scale for the Assessment and Rating of Ataxia; CCMI: cortical cerebral microinfarct.

Table S6. Sensitivity analysis of the association between CCMI and gait speed at the 1-year follow-up (excluding participants with baseline DWI-hyperintense lesions)

	Unadjusted		Model C		Model D	
	β (95% CI)	*p* value	β (95% CI)	*p* value	β (95% CI)	*p* value
**Gait speed at 1-Year follow-up**	-0.22 (-0.34--0.11)	<0.001	-0.17 (-0.25--0.08)	<0.001	-0.16 (-0.24--0.08)	<0.001

Patients without CCMI were used as the reference group in all models. Among the 192 participants included in the baseline analysis, 18 had DWI-hyperintense lesions on baseline MRI. Among the 74 participants with available 1-year follow-up data, five had baseline DWI-hyperintense lesions and were excluded from this sensitivity analysis. The sensitivity analysis therefore included 69 participants: 24 with CCMI and 45 without CCMI.

Model C: Adjusted for age, sex, height, and the corresponding baseline motor outcome.

Model D: Adjusted for age, sex, height, total CSVD burden, and the corresponding baseline motor outcome.

CI: confidence interval; CCMI: cortical cerebral microinfarct; DWI: diffusion-weighted imaging; CSVD: cerebral small vessel disease.

Table S7. Associations between CCMI and motor function at 1-year follow-up (multiple imputation)

	Unadjusted		Adjusted model[a]		
	β/OR (95% CI)	*p* value	β/OR (95% CI)	*p* value	BH-adjusted *p* value
**1-Year Follow-Up Motor Function**					
Gait speed	-0.24 (-0.37--0.11)	<0.001	-0.16 (-0.24--0.08)	<0.001	—
Poor balance[b]	4.80 (1.25-18.43)	0.02	NA	NA	NA
Abnormal gait[c]	4.53 (1.84-11.12)	0.001	1.80 (0.45-7.16)	0.40	0.60
Repeated chair-stand time	3.45 (1.01-5.89)	0.007	1.93 (0.24-3.61)	0.03	0.09

Patients without CCMI were used as the reference group in all models. Gait speed was designated as the primary motor outcome and was therefore not included in the BH adjustment. BH-adjusted *p* values were calculated from the nominal *p* values in the adjusted model (the non-estimable poor-balance test was conservatively assigned *p*=1).

[a]Adjusted for age, sex, height, total CSVD burden, and the corresponding baseline motor outcome.

[b]A score ≤2 on the balance component of the Short Physical Performance Battery. The adjusted estimate was unavailable because of the small number of poor-balance events and model instability.

[c]A score ≥2 on the gait item of the Scale for the Assessment and Rating of Ataxia.

CI: confidence interval; OR: odds ratio; BH: Benjamini–Hochberg; CCMI: cortical cerebral microinfarct; CSVD: cerebral small vessel disease; NA: not available.

Table S8. Estimates of the association between CCMI and gait speed at 1-year follow-up in the primary and sensitivity analyses

Analysis	β[a]	(95% CI)[a]	*p* value[a]
**1-Year Follow-Up Gait speed**			
Complete-case	−0.1578	−0.2374 to −0.0781	<0.001
Multiple-imputation analysis	−0.1574	−0.2368 to −0.0780	0.0002
Excluding baseline DWI-hyperintense lesions	−0.1597	−0.2443 to −0.0752	0.0005

Patients without CCMI were used as the reference group. Estimates are reported to four decimal places to distinguish the small numerical differences across analyses that appeared identical after rounding to two decimal places.

[a]Adjusted for age, sex, height, total CSVD burden, and the corresponding baseline motor outcome.

CI: confidence interval; CCMI: cortical cerebral microinfarct; DWI: diffusion-weighted imaging; CSVD: cerebral small vessel disease.

Supplementary Method S1.

CIBB-CSVD
Motor and Gait Assessment
Protocol

Standardization requirement: All assessments must be administered in the sequence specified in this manual.

1. Gait Speed Test

6 m timed walk within an 8 m walkway
Equipment: A straight, level 8 m walkway and a stopwatch. Mark the walkway at 0 m, 1 m, 7 m, and 8 m. The central 6 m segment (1-7 m) is timed.

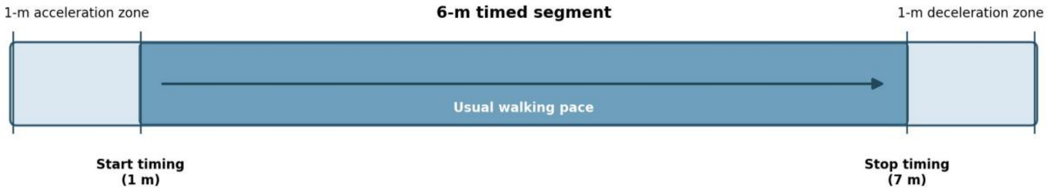

"We will now perform a walking test. Please walk independently without an assistive device."
1. "This is the walkway. Start at the first line, walk beyond the last line, turn around, and walk back at your usual walking pace - similar to the pace you would use when walking to a shop."
2. Each one-way traversal of the walkway constitutes one trial. Complete four trials in total.
3. Demonstrate the walking procedure to the participant.
4. Ask, "Do you have any questions?" Provide further explanation if needed. Then say, "Are you ready? Ready,
go."
5. The first trial is a practice trial and is not timed.
6. Beginning with the second trial, start timing when either foot contacts or crosses the timing start line at 1 m.
7. Stop timing when either foot contacts or crosses the timing end line at 7 m.
8. Record the completion time for each of the three timed trials. Calculate gait speed by dividing 6 m by the mean completion time across the three timed trials.
Safety: The assessor should remain close enough to guard the participant.

2. Standing Balance Test

Side-by-side, semi-tandem, and tandem stands
Eligibility: The participant must be able to stand independently. The assessor may assist the participant in assuming the required starting position.
"We will now begin the assessment. I will first demonstrate several positions, and then I will ask you to try them. If you are unable to complete a position, we will move on to the next assessment. Do you have any questions before we begin?"

A. Side-by-Side Stand

1. "I will first demonstrate the first position."

2. Demonstrate and say, "Place your feet together so that both the heels and toes are touching. Try to maintain this position for 10 seconds."
3. "Keep your heels and toes together and try not to move your feet. Hold the position until I say stop. Do not worry - I will stand beside you to protect you if you become unsteady."
4. Stand beside the participant, assist them into the side-by-side position, and provide adequate guarding to prevent a fall.
5. Once the participant is positioned, ask, "Are you ready?"
6. Allow the participant to stand independently, start timing, and say, "Ready, start."
7. Say, "Stop," and stop timing when the participant completes 10 seconds, grasps the assessor for support because of instability, or moves either foot.
8. If the participant cannot maintain the position for 10 seconds, record the result and discontinue the remaining balance positions.

B. Semi-Tandem Stand
1. "I will now demonstrate the second position."
2. Demonstrate and say, "Place the heel of one foot beside the middle of the other foot and hold this position for 10 seconds. Either foot may be placed in front, according to your preference."
3. "Try not to move your feet and hold the position until I say stop. Do not worry - I will stand beside you to protect you if you become unsteady."
4. Stand beside the participant, assist them into the semi-tandem position, and provide adequate guarding to prevent a fall.
5. Once the participant is positioned, ask, "Are you ready?"
6. Allow the participant to stand independently, start timing, and say, "Ready, start."
7. Say, "Stop," and stop timing when the participant completes 10 seconds, grasps the assessor for support because of instability, or moves either foot.
8. If the participant cannot maintain the position for 10 seconds, record the result and discontinue the tandem stand.

C. Tandem Stand
1. "I will now demonstrate the third position."
2. Demonstrate and say, "Place one foot directly in front of the other, with the heel of the front foot touching the toes of the back foot. Hold this position for 10 seconds. Either foot may be placed in front, according to your preference."
3. "Try not to move your feet and hold the position until I say stop. Do not worry - I will stand beside you
to protect you if you become unsteady."
4. Stand beside the participant, assist them into the tandem position, and provide adequate guarding to prevent a fall.
5. Once the participant is positioned, ask, "Are you ready?"
6. Allow the participant to stand independently, start timing, and say, "Ready, start."
7. Say, "Stop," and stop timing when the participant completes 10 seconds, grasps the assessor for support because of instability, or moves either foot.
8. Record the result and proceed to the next assessment.

Balance positions at a glance

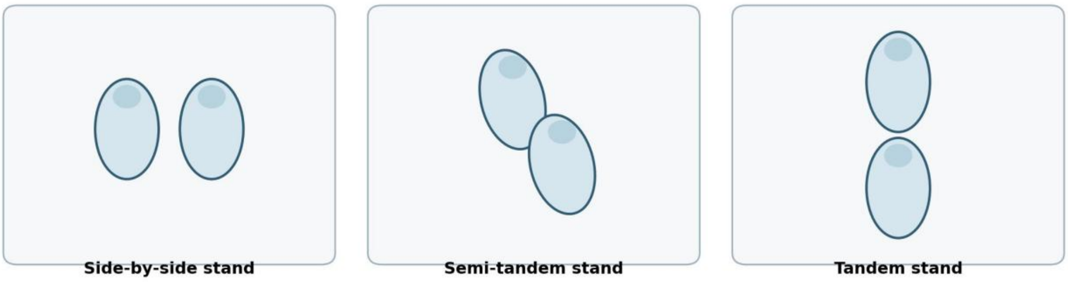

Stopping criterion: For each position, stop timing before 10 seconds if the participant moves either foot or requires external support.

3. SARA Gait Assessment

Ask the participant first to walk at a safe distance parallel to a wall, including a 180° turn, and then to walk 10 consecutive heel-to-toe tandem steps without support.
"Please walk forward heel-to-toe, as in the tandem standing position, for at least 10 consecutive steps."
1. Demonstrate heel-to-toe tandem walking to the participant.
2. Assess the participant while they walk parallel to a wall, complete one 180-degree turn, and perform tandem walking.
3. Assign a score from 0 to 8 using the criteria below. Higher scores indicate more severe gait impairment.

0 Normal walking over 6 m, normal turning, and normal tandem walking; one misstep during tandem walking is permitted.
1 Slight abnormality apparent only during tandem walking (10 consecutive steps).
2 Clearly abnormal; unable to complete more than 10 consecutive tandem steps.
3 Considerable staggering and difficulty turning, but no support is required.
4 Marked staggering; intermittent support from the wall is required.
5 Severe staggering; continuous light support by one arm or use of a cane is required.
6 Able to walk more than 10 m only with strong support (e.g., two specially adapted walking aids or assistance from another person).
7 Unable to walk 10 m even with strong support.
8 Unable to walk even with support.

4. Repeated Chair-Stand Test

Repeated chair-stand assessment of lower-limb function
Equipment: A firm, armless chair with a seat height of approximately 46 cm and a stopwatch.
A. Single Chair-Stand Screening
1. "Next, we will perform the chair-stand test. Please sit on the chair."
2. Demonstrate and explain: "First, fold your arms across your chest. Keep your arms in this position and stand up."
3. Ask the participant to perform one chair stand. If the participant cannot stand with the arms folded across the chest, terminate the test and document the result.
B. Five Repeated Chair Stands

"Now, as before, keep your arms folded across your chest. Stand up fully, sit down, and stand up again as quickly as you safely can. Repeat this five times. I will use a stopwatch to record your completion time."

1. Once the participant is seated securely, ask, "Are you ready?" Then say, "Start," and begin timing.
2. Count each completed repetition aloud.
3. Stop timing when the participant reaches the fully standing position after the fifth rise.
4. If the participant pauses because of fatigue, ask, "Are you able to continue?" If yes, allow the participant to continue and record the total elapsed time. If not, terminate the test and document the result.

Safety and validity: The participant must keep the arms folded across the chest throughout the test. The assessor should remain close enough to guard against loss of balance without providing physical assistance unless needed for safety.

Supplementary Method S2. Multiple imputation of missing 1-year motor outcome data

To assess the potential influence of missing 1-year outcome data, multiple imputation using fully conditional specification was performed under the missing-at-random assumption. Missing values for all four motor outcomes at the 1-year follow-up—gait speed, poor balance performance, abnormal gait, and repeated chair-stand completion time—were imputed. The imputation model included CCMI status, age, sex, education, education-adjusted MoCA score, height, hypertension, diabetes, hyperlipidemia, ever smoking, ever drinking, baseline total CSVD burden, all baseline motor measures, available 1-year motor outcome data, and variables associated with follow-up availability. CCMI status, the exposure of interest, was included as a predictor in the imputation model but was not itself imputed. Continuous outcomes were imputed using linear regression, whereas binary outcomes were imputed using logistic regression. Twenty imputed datasets were generated. The same outcome-specific regression models used in the complete-case analyses were fitted in each imputed dataset, and the resulting parameter estimates and standard errors were pooled using Rubin's rules.
